# Invasive myoepithelial carcinoma ex pleomorphic adenoma of the major salivary gland: two case reports

**DOI:** 10.1186/s12885-016-2871-3

**Published:** 2016-10-28

**Authors:** Takahiro Wakasaki, Marie Kubota, Yutaka Nakashima, Eri Tomonobe, Takenao Mihara, Junichi Fukushima

**Affiliations:** 1 Department of Otorhinolaryngology, Japanese Red Cross Fukuoka Hospital, 3-1-1 Okusu, Miniami-ku, Fukuoka 815-8555 Japan; 2 Department of Pathology, Japanese Red Cross Fukuoka Hospital, 3-1-1 Okusu, Miniami-ku, Fukuoka 815-8555 Japan; 3 Department of Head and Neck Surgery, National Hospital Organization, Kyushu Cancer Center, 3-1-1 Notame, Miniami-ku, Fukuoka 811-1395 Japan

**Keywords:** Case report, Myoepithelial carcinoma, Malignant myoepithelioma, Ex pleomorphic adenoma, Major salivary gland, Ki67 labeling index

## Abstract

**Background:**

Myoepithelial carcinoma (MEC) is a rare salivary gland tumor. Its long-term prognosis remains unknown because of the paucity of reported cases with long-term follow-up. Although some case series exist, the clinical features of MEC vary considerably depending on the site of origin. Therefore, accumulation of these rare cases is important.

**Case presentation:**

Case 1: An 89-year-old man presented with a 10-year history of a mass originating from the right parotid gland and involving the neck. The mass grew rapidly for 3 months, reaching approximately 8 cm. There was no facial paralysis. MEC ex pleomorphic adenoma (PA) was suspected. Superficial parotid gland resection was performed in 2013; the tumor grade was pT3N0M0, and the resection margins were free of carcinoma. Because of several high-risk factors for metastasis (i.e., invasive carcinoma ex PA, high MIB1 index, and mutant p53 protein positivity), radiotherapy and chemotherapy were recommended as adjuvant therapy. Although the patient refused adjuvant therapy, he was recurrence-free at 36 months after surgery.

Case 2: A 54-year-old woman presented with a >10-year history of a right submandibular mass, which grew rapidly for 1 year, reaching approximately 6 cm. Preoperative diagnosis was PA of the right submandibular gland. Submandibular gland resection was performed in 2013. Pathological analysis revealed invasive MEC ex PA, pT3N0M0; in addition, the carcinoma portion had an extra capsule and had invaded the platysma muscle close to the margin. An MIB1 index of 40 % and mutant p53 protein positivity indicated a high risk for metastasis. Additional resection and right neck dissection revealed no residual carcinoma. The patient refused adjuvant chemotherapy. One year after surgery, metastasis to the right pulmonary hilar node and both lungs were detected. Chemotherapy prevented recurrent growth of the lesion and extended survival. The patient was alive with cancer 30 months after the first surgery.

**Conclusions:**

High expression of the Ki67 labeling index might reflect prognosis of these cases. Chemotherapy for distant metastasis was effective, as expected. Further accumulation of cases and long follow-up data are needed to elucidate the pathophysiology and prognosis of MEC ex PA.

## Background

Myoepithelial carcinoma (MEC) is a rare salivary gland tumor, accounting for 0.1–0.45 % of all salivary gland tumors [1, 2]. MEC generally arises from the parotid gland (29–82 % of all cases) [2] and rarely from the submandibular gland, minor salivary glands, or the upper respiratory tract [2–4]. Other than the head and neck, MEC has been reported in the lung, breast, bronchus, mediastinum, and others sites [2, 5, 6]. Because of its rarity, the clinical features and pathophysiology of MEC are not well characterized; most articles describing MEC are case reports. According to a few large case series (>10 cases), the mortality rate of MEC is 20–73 % [2, 7–9]. Regional node involvement is reported in 0–41 % of cases [3, 7]. A distant metastasis rate is approximately 6.8–47 %, most commonly to the lung, brain, spine, and skin [2]. These values may differ considerably depending on the site of origin of MEC. Furthermore, the efficacy of radiation therapy and/or chemotherapy for MEC remains unclear because of the varied clinical courses. Preoperative diagnosis of salivary gland tumors is often difficult because of the overlap in histopathological characteristics. Moreover, even if the pathological diagnosis is established postoperatively, there is no consensus on appropriate adjunctive treatment strategies because of the rarity of salivary gland tumors, such as MEC [10]. Although high-risk factors for MEC of the major salivary gland have been gradually proposed, the accumulation of a greater number of cases is necessary to determine effective treatment strategies.

We present two cases of MEC ex pleomorphic adenoma (PA) of the major salivary gland. The prognosis of these cases was reflected by an unusually high Ki67 labeling index. We also summarize the risk factors for MEC ex PA with a mini-review of the literature.

## Case presentation

**Fig. 1:**
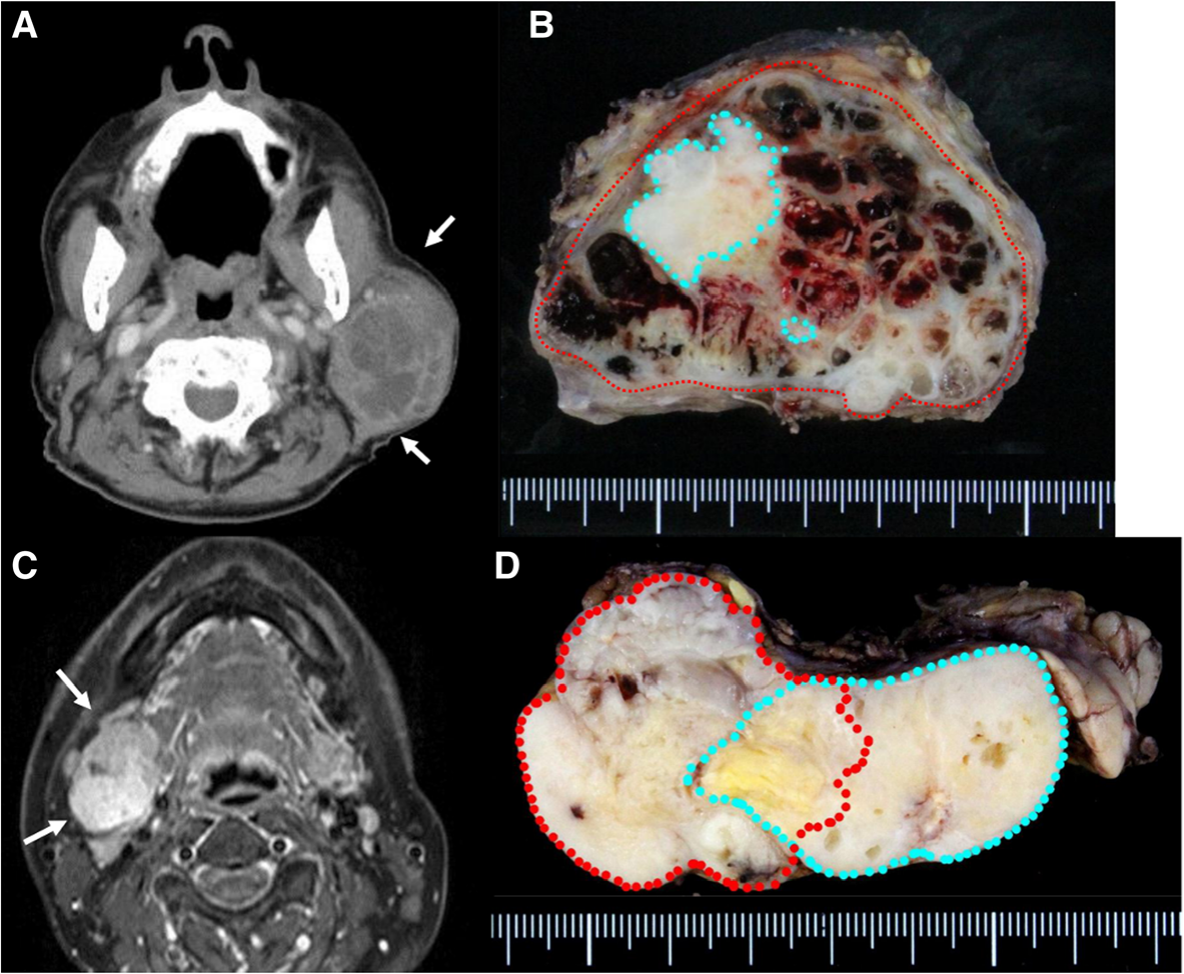
Enhanced CT revealed a large left neck mass with partial enhancement. Necrosis of the inner tumor was suspected. **a** Macroview of the tumor: PA was completely surrounded by MEC. *Red line*, cancer; *blue line*. PA, **b** MRI revealed a mass with bumpy surface in the right submandibular gland with a partially unclear border. **c** Macroview of the tumor: MEC existed outside of PA. *Red line*, cancer; *blue line*, PA

**Fig. 2:**
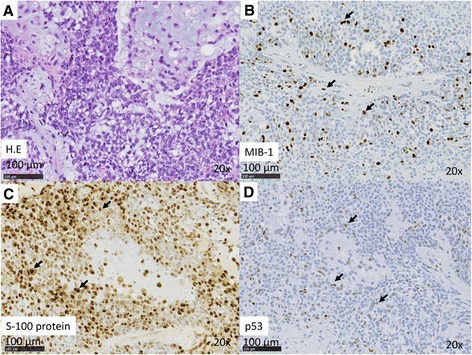
(**a**–**d**) Case 1. Histopathologic features of MEC. **a** H&E stain. **b** IHC analysis reveals Ki67 positivity in nuclei. The MIB index was approximately 20 %. **c** IHC analysis revealed S-100 positivity in the cytoplasm. **d** IHC analysis revealed p53 positivity in the nuclei

Case 1: An 89-year-old man presented to us in 2013 with a 10-year history of a mass originating from the right parotid and involving the neck. The mass grew rapidly for 3 months before the patient was examined. At presentation, the diameter of this rubbery hard mass was approximately 8 cm. There was no facial paralysis. Computed tomography (CT) showed an irregular mass of the right parotid gland with partially defined margins and no lymph node metastases (Fig. 1a). Magnetic resonance imaging (MRI) was not performed because of presence of metal implants for a left leg fracture. Fine-needle aspiration cytology (FNAC) of the parotid mass indicated PA. We suspected that the mass was a malignant tumor, particularly a carcinoma ex PA. Therefore, superficial parotid gland resection was performed in March 2013. Intraoperatively, the facial nerve was found attached to the tumor, although the tumor was resected without sacrificing the facial nerve. Pathological analysis of the tumor revealed MEC ex PA (pT3N0M0), MEC component was clear cell type, and the resection margin was free of carcinoma. The histological grade was low according to the grading system of Savera [11]. (Fig. 1b, Fig. 2a–d). PA was completely surrounded by carcinoma that indicated pathological invasiveness [12]. Immunohistochemical (IHC) analysis was positive for S-100 protein, pan-cytokeratin (AE1/AE3), smooth muscle actin, p63, and p53 (Fig. 2c, d). Moreover, the nucleus was moderately positive for mindbomb E3 ubiquitin protein ligase 1 (MIB-1) (active area, 20 %) (Fig. 2b). The patient was considered as high risk because of the findings of invasive MEC ex PA and mutant p53 protein positivity; thus, radiotherapy for the locoregional area was recommended. However, the patient refused adjuvant therapy because of his age. At the 36-month postoperative follow-up, he was still without recurrence.

**Fig. 3:**
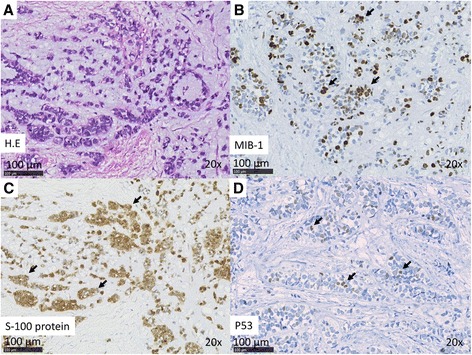
(**a**–**d**) Case 2. Histopathologic features of MEC. **a** H&E stain. **b** IHC analysis revealed Ki67 positivity in the nuclei. The MIB index was approximately 40 %. **c** IHC analysis revealed S-100 positivity in the cytoplasm. **d** IHC analysis revealed p53 positivity in the nuclei

Case 2: A 54-year-old woman presented to us in 2013 with a >10-year history of right submandibular lesion. The growth of the mass was slightly faster 1 year before presentation. At presentation, the elastic hard mass was approximately 6 cm in diameter, and there was no facial paralysis. MRI showed an irregular mass in the right submandibular gland with partially defined margins (Fig. 1c). There was no indication of lymph node metastases on ultrasonography and MRI. Both dynamic MRI and FNAC from the submandibular mass indicated PA. However, we also suspected carcinoma ex PA because of the rapid growth of the tumor. Submandibular gland resection was performed in July 2013. During resection, part of the platysma muscle with suspected adhesion was removed, and the mass was excised along the capsule. The marginal mandibular branch of the facial nerve was preserved because it was not adhered to the tumor. Pathological analysis of the tumor revealed invasive MEC ex PA, pT3N0M0, whereas the carcinoma portion had an extracapsular spread into and invaded the platysma muscle. The excision stump was close to the carcinoma portion (Fig. 1d, Fig. 3a–d). The MEC component comprised clear cell type. The histological grade was high [11]. IHC analysis was positive for S-100 protein, AE1/AE3, and p53 (Fig. 3c, d). Moreover, the nucleus was strongly positive for MIB1 (active area, 40 %) (Fig. 3b), whereas IHC analysis was negative for p63. Because of the close margin, right neck dissection (levels I–III) and additional resection of the submandibular tissue, including the platysma muscle around the tumor, was performed in August 2013. Pathological analysis revealed no regional metastases or residual carcinoma in the additional resected tissue. Because of several positive risk factors (invasive MEC ex PA, high MIB1 index, and mutant p53 protein positivity), radiotherapy and chemotherapy were recommended as adjuvant therapy. At that time, she refused additional therapy and was followed as an outpatient. One year after surgery, distant metastasis to the right pulmonary hilar node and lungs was revealed by follow-up CT and 2-deoxy-2-[fluorine-18]fluoro-D-glucose positron emission tomography integrated with CT. There were typical multiple metastases to both lungs, with a maximum tumor diameter of 3 cm. We diagnosed the lesions on observation of distant metastases from the known salivary gland malignancy according to the typical CT imaging and the clinical course of the enlarging multiple nodules at the peripheral lung field and right hilar node. Eight cycles of chemotherapy with cyclophosphamide 500 mg/m^2^, cisplatin 50 mg/m^2^, and adriamycin 50 mg/m^2^ were intermittently initiated [13]. No serious adverse events were observed during chemotherapy. The lung metastases were controlled; therefore, at the follow-up 30 months after the first surgery, she was still alive with cancer.

## Discussion

We experienced two cases of MEC ex PA of the major salivary gland. The MEC in Case 2 presented with a clinically aggressive behavior compared to Case 1, which was also indicated by the MIB1 index score. However, chemotherapy for Case 2 could contribute to an improvement in the quality of life in the future, for example, prevention from dyspnea or pleural effusion, and extended survival.

Precise preoperative diagnosis of MEC ex PA is almost impossible, and risk factors for prognosis remain uncertain [2]. Symptoms of MEC are similar to those of other salivary gland tumors, i.e., a mass with or without pain, facial palsy, and fixation of the mass to the underlying structures [3]. As with other high-grade malignancies, the usefulness of FNAC is limited for preoperative diagnosis of salivary gland carcinoma [14]. The sensitivity of FNAC for the preoperative diagnosis of carcinoma ex PA is only 29–54 % [15, 16]. In our patients, it was also not possible to arrive at a preoperative diagnosis, although the preoperative clinical course of both suggested salivary gland tumor.

Pathological analysis is performed to identify risk factors and plan adjuvant therapies. However, specific risk factors and the efficacy of postoperative therapy for MEC ex PA remain unknown. Past reports and reviews have suggested candidate markers to predict the prognosis of MEC (Table 1). MECs originating from a minor salivary gland reportedly have relatively better prognoses [17]. However, whether prognosis for MEC ex benign tumor, particularly PA, is better than for de novo MEC remains controversial. Some reports claim that a de novo mass is highly malignant with high relapse rates, whereas others report the contrary [3, 8, 18]. Thus, prognosis of MEC ex PA remains unknown [15]. The presence of spindle cells may correlate with aggressive behavior, nodal metastasis, and distant metastasis [3]. Most tumors that display histologic aggressiveness behave adversely [2]. Some reports have described the relationships between IHC findings and the clinical course. Overexpression of Ki67, which reflects cell proliferation, might be a useful marker of high risk of prognosis in MEC. Nagao et al. reported that four of five patients with high expression of nuclear Ki67 (MIB1-index >30 %) died of the disease [1]. They also described that the mean MIB1-index of fatal and favorable cases was 51.6 % (SD: 11.4 %) and 25.7 % (SD: 13.2 %), respectively. We understand that the definition about high MIB1 index of the MEC in the salivary gland remains unknown. However, we think that a value of 30–40 % greater than the MIB1 index might support distinguishing of the higher grade carcinoma in MEC. Therefore, we think that case 2 is of a high grade malignancy. Mutant p53 or p63 protein positivity has been reported as a risk factor of various cancers and presents a potential marker for a high risk of malignancy and prognosis for MEC [1, 5]. The MIB1 index for Case 2 was 40 %, which indicated a high risk for malignancy and might reflect early distant metastasis, compared to Case 1.

**Table 1:** Summary of the suggestive risk factor for poor prognosis in MEC

High risk	
Histological findings: Spindle cell type [3]	
IHC for p53, p63 is positive, Ki67 labeling index is high [1, 3, 5]	
Clinical stage, size, extensive invasion into the surrounding tissue, perineural invasion [2, 12, 19]	
Low risk	
Site of origin: a minor salivary gland [17]	
Histological findings: Clear cell component [20]	
Unknown or Equivalent or controversial	
MC derived from benign tumor, PA, or myoepithelioma [2, 21]	

A list of the treatment modality and outcomes in the MEC of the salivary gland from previous articles is summarized in Table 2. In most reported cases, the initial strategy is tumor resection [1, 2]. Because MEC is a highly malignant, radical resection is most desirable, if possible. Although there is currently no consensus on strategies to preserve the facial nerve, with high-grade cancer and cases of local recurrence, the cancerous portions of the salivary gland should be resected with facial nerve sacrifice [3, 17]. Regarding neck dissection, there is little information about the risk factors of regional lymph node metastasis; therefore, elective neck dissection should be determined on a case-by-case basis.

**Table 2:** Summary of the treatment modality and outcomes in MEC of salivary gland among the larger series in the literature

Article/year	Number o. of patients	Treatment	Recurrence	Mortality by tumor	Time of Follow-up
Kane et al./2010 [3]	44	Radical Surgery (44) Radiation Therapy (12)	18/44	NA	1y–10y
Yu et al./2003 [7]	27	Radical Surgery (26) Radiation Therapy (12)	14/27	10/27	9 m–17y
Savera et al./2000 [11]	25	Radical Surgery (25) Radiation Therapy (6) Chemotherapy (2)	8/17^a^	5/17	6 m–8y
Santos et al./2016 [21]	19	Radical Surgery (19) Radiation Therapy (9) Chemotherapy (1)	7/19	4/19	2 m–15y
Jiang et al./2012 [5]	11	Radical Surgery (11) Radiation Therapy (9) Chemotherapy (2)	6/11	4/11	1y–6y
Dipalma et al./1993 [8]	10	Radical Surgery (10)	8/10	2/10	1y–35y

Modification and Reference from S. Vilar-Gonzalez et al. 2015 Clin Transl Oncol

^a^8 of 25 patients were lost to follow-up

Although the efficacy of radiation therapy for MEC ex PA remains unknown, adjuvant radiotherapy with or without chemotherapy is suggested for high-grade malignancies with residual tumor/close margin, neural/perineural invasion, soft-tissue extension, lymph node involvement, or lymphatic/vascular invasion, as well as after salvage surgery for recurrent tumors [12, 19]. Moreover, the efficacy and the optimal timing of chemotherapy for MEC ex PA too remain unclear. In most cases, chemotherapy was administered concurrently with radiotherapy as an adjuvant therapy or solely for distant metastasis. Chemotherapy might be useful for patients with distant metastases, as illustrated by Case 2 in this study, in which chemotherapy for pulmonary metastasis appeared to help control problems related to the quality of life from occurring in the future and extend survival.

## Conclusions

We reported two rare cases of MEC ex PA originating in the major salivary gland. High expression of the Ki67 labeling index might reflect prognosis of these cases, and chemotherapy for distant metastasis was effective, as expected. Further accumulation of cases and long follow-up data are needed to elucidate the pathophysiology and prognosis of MEC. We will continue to follow these patients to obtain long-term survival data.

## Abbreviations

CA: Carcinoma
CT: Computed tomography
ex PA: ex pleomorphic adenoma
FNAC: Fine-needle aspiration cytology
IHC: Immunohistochemical
MEC: Myoepithelial carcinoma
MRI: Magnetic resonance imaging
